# The adjusted impact of different severities of acute exacerbations and medications on the risk of developing dementia in COPD patients

**DOI:** 10.1186/s12890-023-02386-8

**Published:** 2023-03-29

**Authors:** Kuo-Hua Chia, Yao-Yuan Chang, Tren-Yi Chen, Pei-You Hsieh, Cheng-Chieh Huang, Tsung-Han Lee, Cheng Hsu Chen, Wen-Liang Chen, Chu-Chung Chou, Yan-Ren Lin

**Affiliations:** 1 Department of Emergency and Critical Care Medicine, Changhua Christian Hospital, Changhua, Taiwan; 2 Department of Emergency Medicine, Yuanlin Changhua Christian Hospital, Changhua, Taiwan; 3 Department of Biological Science and Technology, National Yang Ming Chiao Tung University, Hsinchu, Taiwan; 4 Department of Post Baccalaureate Medicine, College of Medicine, National Chung Hsing University, Taichung, Taiwan; 5 School of Medicine, Kaohsiung Medical University, Kaohsiung, Taiwan; 6 School of Medicine, Chung Shan Medical University, Taichung, Taiwan

**Keywords:** Bronchodilators, COPD, Dementia, Corticosteroids

## Abstract

**Background:**

Although a relationship between chronic obstructive pulmonary disease (COPD) and dementia has been reported, the initial severity upon emergency department (ED) visits and the medications used have not been well evaluated as risk factors for increased dementia occurrence. We aimed to analyze the risks of dementia development over 5 years among patients with COPD compared to matched controls (primary) and the impact of different severities of acute exacerbations (AEs) of COPD and medications on the risk of dementia development among COPD patients (secondary).

**Method:**

This study used the Taiwanese government deidentified health care database. We enrolled patients during the 10-year study period (January 1, 2000, to December 31, 2010), and each patient was followed up for 5 years. Once these patients received a diagnosis of dementia or died, they were no longer followed up. The study group included 51,318 patients who were diagnosed with COPD and 51,318 matched (in terms of age, sex, and the number of hospital visits) non-COPD patients from the remaining patients as the control group. Each patient was followed up for 5 years to analyze the risk of dementia with Cox regression analysis. Data on medications (antibiotics, bronchodilators, corticosteroids) and severity at the initial ED visit (ED treatment only, hospital admission, or ICU admission) were collected for both groups, as well as demographics and baseline comorbidities, which were considered confounding factors.

**Results:**

In the study and control groups, 1,025 (2.0%) and 423 (0.8%) patients suffered from dementia, respectively. The unadjusted HR for dementia was 2.51 (95% CI: 2.24–2.81) in the study group. Bronchodilator treatment was associated with the HRs, especially among those who received long-term (> 1 month) treatment (HR = 2.10, 95% CI: 1.91–2.45). Furthermore, among 3,451 AE of COPD patients who initially visited the ED, patients who required ICU admission (n = 164, 4.7%) had a higher risk of dementia occurrence (HR = 11.05, 95% CI: 7.77–15.71).

**Conclusion:**

Bronchodilator administration might be associated with a decreased risk of dementia development. More importantly, patients who suffered AEs of COPD and initially visited the ED and required ICU admission had a higher risk of developing dementia.

**Supplementary Information:**

The online version contains supplementary material available at 10.1186/s12890-023-02386-8.

## Introduction

The outcomes of patients with severe COPD are poor. It was estimated that at least 3 million deaths are caused by COPD annually (which is responsible for almost 5% of all deaths worldwide)[1–4]. The incidence rate of COPD increased with age among people of both sexes and decreased in all age groups from 1990 to 2017.[5] COPD has been associated with several risk factors, including air pollution (indoor or outdoor), tobacco smoke, infections and allergic diseases.[6–8] Therefore, medical resource expenditures for handling COPD (and related comorbidities) have gradually become a national burden, especially in developed countries.[9, 10] The comorbidities of COPD are diverse. In addition to increasing the risk of carcinogenicity (lung cancer), COPD poses a serious risk to the central nervous system.[11, 12] The long-term hypoxia, hypercapnia, and increased inflammatory cytokines that are typical characteristics of COPD contribute to the development of degenerative brain diseases (including structural changes in the brain and reduced white matter integrity).[11, 13] Furthermore, some studies have noted that patients with COPD are at high risk of developing dementia. In fact, people of different ages are at significant risk of dementia. Unlike older COPD patients, some studies have reported that patients younger than 64 years have a higher risk of dementia. COPD-related risk factors affect the age of onset, with a greater risk among younger individuals, resulting in a higher risk of dementia.[5, 14] Patients with COPD have a higher risk of cognitive dysfunction, especially verbal memory impairment and learning impairment. [15, 16]

Although the association between COPD and dementia has been well studied, the COPD stabilization treatment strategy that would further decrease (or even increase) the risk of developing dementia has never been well addressed. In addition, the control of external/internal predisposing factors for COPD (i.e., smoking, the prevention of allergies or infections)[17, 18] and several categories of medications used for stabilizing acute exacerbations (AEs) of COPD and their impacts on patient risk of dementia have not been well discussed. Steroids, bronchodilators, and antibiotics are the most useful medications recommended for short-term or even long-term treatment of COPD[19–21]. Some previous studies reported that antibiotics used over 91 days, long-term use of inhaled corticosteroids and the bronchodilator-related “anticholinergic burden” might induce cognitive problems.[22–24] Therefore, we suspect that the risk of developing dementia could also be modulated by these medications. However, this association has never been clearly demonstrated, especially regarding short-term or long-term medication treatment.

Moreover, since the major symptoms of AEs of COPD are related to dyspnea or hypoxia and are sometimes life-threatening, up to 65% of patients choose to visit the emergency department (ED) first by ambulance for initial stabilization.[25, 26] Some previous studies have mentioned that hypoxia can alter neuronal cell dysregulation and neuroinflammation.[27, 28] Therefore, we suspect that more severe AEs might be related to worse hypoxia and further induce neuronal dysfunction. Appropriate medication treatments for COPD/AEs might reduce the risk of dementia. We suspect that ED severity classification might also be a risk factor for dementia occurrence, but this relationship is also not clear. In this study, we aimed to analyze the risks of dementia development over 5 years among patients with COPD compared to matched controls (primary) and the impact of different severities of AEs of COPD and medications on the risk of dementia development among COPD patients (secondary).

## Materials and methods

### Database

The database that we used in this study was provided by the Taiwanese government (National Health Insurance Program, Ministry of Health and Welfare). This database includes the medical information of patients (i.e., diagnoses, treatments, medications, outcomes) and covers almost 100% of the population in Taiwan. All information in this database (and in this study) was deidentified secondary data. Personal patient information could not be identified or followed up. This study was conducted according to the regulations of the Taiwanese government and the institutional review board of the study hospital (IRB No. 181,109).

### Setting

We enrolled patients during the 10-year study period (January 1, 2000, to December 31, 2010), and each patient was followed up for 5 years. Once these patients received a diagnosis of dementia or died, they were no longer followed up. Patients were selected from this database and divided into two groups (the study and control groups). We stopped collecting medication data once the patients received a diagnosis of dementia. The matched controls were only followed up to the point that the COPD patients received a diagnosis of dementia. In the study (with COPD) and control (non-COPD) groups, each patient was followed up for 5 years to analyze the risk factors for the development of new-onset dementia. All patients were followed up initially from a definite date. For the study group, the date was the first diagnosis of COPD. For the control group, the date was the same month as for patients in the study group.

### Selection methods

#### Assessment limitations of the deidentified database

The deidentified data were limited to only scientific research (from January 1, 2000, to December 31, 2010). Therefore, ICD-9-CM (not ICD-10-CM) codes were used in this study.

#### Inclusion criteria for the study group (with COPD)

During the study period, patients who were primarily diagnosed with COPD ICD-9-CM codes (490–492 and 496) in the ED or outpatient department (OPD) or upon hospital admission were defined as COPD patients and classified as the study group. Since we attempted to analyze the association between COPD and dementia, we only included the “first diagnosis” of COPD. The “first diagnosis” was determined according to their “first-time” ICD-9-CM codes for COPD, which were reported by their doctors using the National Health Insurance Program (clinical diagnosis).

#### Exclusion criteria

In both the study and control groups, patients were excluded due to the following criteria (Supplementary Material 1):

1. Patients aged younger than 18 years.
2. Patients with any history of COPD, asthma, or chronic bronchitis (if any patient had these histories and could not be followed up on their “first diagnosis” during the study period, they were excluded).
3. Patients with any history of dementia, including other forms of dementia (Alzheimer’s disease, vascular dementia, Lewy body dementia, Parkinson’s disease-related dementia and frontotemporal dementia).
4. Patients with other long-term degenerative brain diseases or impaired brain cognitive function (including prion disease, Parkinson’s disease without dementia, Parkinson-plus syndromes, amyotrophic lateral sclerosis/motor neuron disease, and brain encephalopathy caused by drugs or substances).
5. Patients with incomplete medical records or those who could not be followed up (canceled insurance).

#### Dementia (primary outcome)

Dementia was defined as a major diagnosis with ICD-9-CM codes 290.0 (senile dementia uncomplicated), 290.1 (presenile dementia), 290.4 (vascular dementia) and 331.0. (Alzheimer’s disease). However, we did not use codes 294.1 (dementia in conditions classified elsewhere) and 294.2 (dementia, unspecified, without behavioral disturbance) to confirm the diagnosis of dementia. In this database, some diagnoses were mixed with other nonspecific codiagnoses, including delirium or other psychosis problems. To ensure accuracy, we did not include code 294.1 or 294.2. In Taiwan, the diagnosis of dementia is usually made by psychiatrists or neurologists by using standard checklists. (i.e., Mini-Mental Status Examination, Clinical Dementia Rating).

#### Selection methods for the control group

The study group ultimately included 51,318 patients with COPD. We randomly selected patients for the control group from the remaining people in the database. Each patient in the control group was matched individually (one-to-one by age, sex, the number of hospital visits, and the length of follow-up). The control group also ultimately comprised 51,318 patients. The total population in this study was 102,636. We used post hoc power analysis to calculate the power and study parameters population incidence = 6.1%, study group incidence = 5.1%, subjects = 51,318 and alpha = 0.05, and post hoc power = 100%. [29]

### Statistical analysis

The data were analyzed using SPSS software (for Windows, Version 15.0, SPSS Inc., Chicago, IL). We used descriptive analysis, the chi-squared test, Cox regression analysis and Kaplan‒Meier curves to analyze group differences and differences in the risk of new-onset dementia between the study and control groups. The demographics of the study and control groups were reported as percentages or means ± standard deviations (SDs), and the chi-squared test was used to compare the differences between the two groups. The demographics evaluated included age (< 30, 30 to 50, 51 to 70, > 70 years), the number of hospital visits during the study period, monthly income and baseline comorbidities (diabetes mellitus, hypertension, renal failure, liver cirrhosis, stroke and osteoporosis). These comorbidities are common in older populations and might be associated with the risk of dementia.[30–32].

Information regarding medications (corticosteroids, antibiotics, bronchodilators) that are used for treating COPD and associated complications was also obtained.[33, 34] Medications were not included in the analyses if they were not used for the purpose of treating COPD and associated complications. For example, corticosteroids for long-term treatment of psoriasis were not included. Oral and injected formulations and oral, injected, and inhaled formulations of antibiotics and corticosteroids, respectively, were included (topical formulations were not included). The bronchodilators included were short/long-acting beta-adrenergic agonists, anticholinergic drugs and theophylline (oral, injected, and inhaled formulations). Some central nervous system stimulant drugs that have similar bronchodilating effects (i.e., methamphetamine and cocaine) were not considered bronchodilators in this study.

#### The definitions of short- and long-term medications

Long-term treatment in this study was defined as continuous and regular medication use for over 1 month. Short-term treatment was defined as medication use for one month or less. The patients might have received multiple short courses of medication. However, we did not count each prescription as a separate data point. Treatment was based on the first prescription that met our short-term (or long-term) criteria. Cox regression analysis was used to analyze the risk of dementia in short-term and long-term treatment models (Model 1: adjusted for antibiotics; Model 2: adjusted for bronchodilators; Model 3; adjusted for corticosteroids; Model 4: adjusted for all drugs) after considering other potential confounding factors (including age, sex, income, demographics, and baseline comorbidities).

#### The definition of COPD AEs severity

In addition, Cox regression analysis was used to compare the risks among different severities of AEs of COPD for patients who initially visited the ED. The severity of AEs of COPD was classified as the final required treatment for patients, including ED treatment only, hospital admission (ward) and ICU admission. For example, if a patient was initially admitted to the ward but was then referred to the ICU as their condition worsened, the patient was classified as an ICU admission. Some patients with AEs of COPD required multiple admissions. We obtained their first-time ED visits for AEs of COPD.

## Results

### Demographics of the study and control groups

The characteristics and baseline comorbidities of the study (with COPD; n = 51,318) and control (without COPD; n = 51,318) groups are presented in Table 1. Among the COPD patients, the 30- to 50-year-old age group was the most predominant (42.8%). The study group had a higher prevalence of baseline comorbidities (including diabetes mellitus, hypertension, and renal failure) (all *p* < 0.001).

**Table 1:** Characteristics and baseline comorbidities among patients with COPD and controls

	COPD group (n = 51,318)		Control group (n = 51,318)		
	No.	%	No.	%	*p* value
Sex					1.000
Male	25,835	50.3	25,835	50.3	
Female	25,483	49.7	25,483	49.7	
Age group (y/o)					1.000
< 30	12,493	24.3	12,493	24.3	
30 to 50	21,958	42.8	21,958	42.8	
51 to 70	12,666	24.7	12,666	24.7	
> 70	4,201	8.2	4,201	8.2	
The number of hospital visits during the follow-up period (mean ± SD)	14.6 ± 8.8		14.6 ± 8.8		1.000
Monthly income (USD$)^a^					0.014
< 600	21,026	41.0	21,472	41.8	
601 ~ 1000	21,073	41.1	20,847	40.6	
> 1000	9,219	18.0	8,999	17.5	
Baseline comorbidities					
Diabetes mellitus ^a^	3,607	7.0	3,246	6.3	< 0.001
Hypertension ^a^	6,732	13.1	5,617	10.9	< 0.001
Renal failure ^a^	498	1.0	377	0.7	< 0.001
Liver cirrhosis ^a^	368	0.7	244	0.5	< 0.001
Stroke ^a^	735	1.4	549	1.1	< 0.001
Osteoporosis ^a^	1,359	2.6	1,079	2.1	< 0.001
AEs of COPD and admission to the ED	3,451	6.7	-	-	
Severity of AEs of COPD					
ED treatment only	2,003	57.9	-	-	
Hospital admission	1,284	37.1	-	-	
ICU admission	164	4.7	-	-	
Short-term medications^b^					
Corticosteroids	3,427	6.7	-	-	
Antibiotics	6,510	12.7	-	-	
Bronchodilators	10,856	21.2	-	-	
Long-term medications^c^					
Corticosteroids	7,066	13.8	-	-	
Antibiotics	17,141	33.4	-	-	
Bronchodilators	14,865	29.0	-	-	

^a^Significant differences

^b^Short-term treatment was defined as medication use for one month or less

^c^Long-term treatment in this study was defined as continuous and regular medication use for over 1 month

### Medications and AEs of COPD

Among the 51,318 COPD patients, 21.2% (n = 10,856**)** and 29% (n = 14,865) were treated with short-term and long-term bronchodilators, respectively. Detailed information on the medications is shown in Table 1. In addition, 3,451 patients (6.7%) suffered AEs of COPD and were admitted to the ED. Most AEs (n = 2003, 57.9%) required only short-term treatment in the ED. However, 1,284 (37.1%) and 164 (4.7%) patients required hospital and ICU admission, respectively.

### Dementia likelihood based on the crude HR

#### Adjustment for demographics and baseline comorbidities

During the 5-year follow-up period, 1,025 (2.0%) and 423 (0.8%) patients developed new-onset dementia in the study and control groups, respectively. Cox proportional hazards analysis showed that the study group had a crude HR 2.51 times greater than that of the control group (HR: 2.51, 95% CI: 2.24–2.81). Furthermore, after adjusting for the demographics and baseline comorbidities of patients, the HR of dementia in the study group was still higher than that in the control group (HR: 2.42, 95% CI: 2.16–2.71) (Table 2). The average length of time until dementia diagnosis (n = 1,025) for COPD patients (n = 51,318) was 2.6 ± 1.5 years (964.5 ± 538.4 days).

**Table 2:** Crude HRs for the presence of new-onset dementia among patients with COPD and controls

Development of dementia during the 5-year follow-up period	Total sample (n = 102,636)		COPD group (n = 51,318)		Control group (n = 51,318)	
	No.	%	No.	%	No.	%
Yes	1,488	1.4	1,025	2.0	423	0.8
No	101,188	98.6	50,293	98.0	50,895	99.2
Crude HR (95% CI)	-		2.51^a^ (2.24–2.81)		-	
Adjusted HR (95% CI)	-		2.42^b^ (2.16–2.71)		-	

^a^ The crude HR was calculated using a Cox proportional hazards model stratified by age, sex and the year of index hospitalization

^b^HR adjusted for demographics and baseline comorbidities (i.e., diabetes, hypertension, renal failure, liver cirrhosis, stroke and osteoporosis)

#### Adjustment for medications

The antibiotics, bronchodilators and steroids used to control COPD may have been associated with the risk of dementia (Table 3). Among them, bronchodilators were the most common drugs. The HR changed to 2.12 (95% CI: 2.05–2.62) when bronchodilators were administered for short-term treatment. Moreover, the HR changed to 2.10 (95% CI: 1.91–2.45) when bronchodilators were administered for long-term treatment.

**Table 3:** The adjusted effect of medications on dementia

Short-term medications			Long-term medications		
Variables	Occurrence of new-onset dementia		Variables	Occurrence of new-onset dementia	
	HR^a^	95% CI		HR^a^	95% CI
Model 1			Model 1		
COPD group	2.35	2.09–2.64	COPD group	2.28	2.03–2.56
Control group^b^	1.00	1.00	Control group^b^	1.00	1.00
Model 2			Model 2		
COPD group	2.12	2.05–2.62	COPD group	2.10	1.91–2.45
Control group^b^	1.00	1.00	Control group^b^	1.00	1.00
Model 3			Model 3		
COPD group	2.40	2.14–2.70	COPD group	2.34	2.08–2.62
Control group^b^	1.00	1.00	Control group^b^	1.00	1.00
Model 4			Model 4		
COPD group	2.31	2.04–2.61	COPD group	2.15	1.91–2.43
Control group^b^	1.00	1.00	Control group^b^	1.00	1.00

The HR was calculated using a Cox proportional hazards model stratified by age, sex and the year of index hospitalization

^a^ All HRs were adjusted for demographics and baseline comorbidities (i.e., diabetes, hypertension, renal failure, liver cirrhosis, stroke and osteoporosis)

^b^Reference group

Model 1: Adjusted for the use of antibiotics

Model 2: Adjusted for the use of bronchodilators

Model 3: Adjusted for the use of corticosteroids

Model 4: Adjusted for the use of all drugs (antibiotics, bronchodilators, corticosteroids). ^a^

#### Adjustment for the severity of AEs of COPD

The severity of AEs of COPD was clearly associated with the HR of dementia. The HRs were highest for patients who required ICU admission (HR: 11.05, 95% CI: 7.77–15.71), followed by those who required hospital admission (HR: 8.52, 95% CI: 7.23–10.03) and ED visits only (HR: 2.11, 95% CI: 1.68–2.65) (Table 4).

**Table 4:** The adjusted effect of dementia development for different severities of COPD

	Patients who suffered AEs of COPD and were admitted to the ED in the study group (n = 3,451)							
Development of dementia during the study period	ED treatment only (n = 2,003)		Hospital admission (n = 1,284)		ICU admission (n = 164)		Controls (n = 51,318)	
	No.	%	No.	%	No.	%	No.	%
Yes	80	4.0	173	13.5	32	19.5	423	0.8
No	1,923	96.0	1,111	86.5	132	80.5	50,895	99.2
Crude hazard ratio^ab^ (95% CI)	2.11*(1.68–2.65)		8.52*(7.23–10.03)		11.05*(7.77–15.71)		-	

^a^ The crude hazard ratio was calculated by a stratified Cox proportional hazards model (stratified by age, sex, and the year of index hospitalization)

^b^ Compared with the control group

*p < 0.001

#### Dementia-free survival curves

**Fig. 1:**
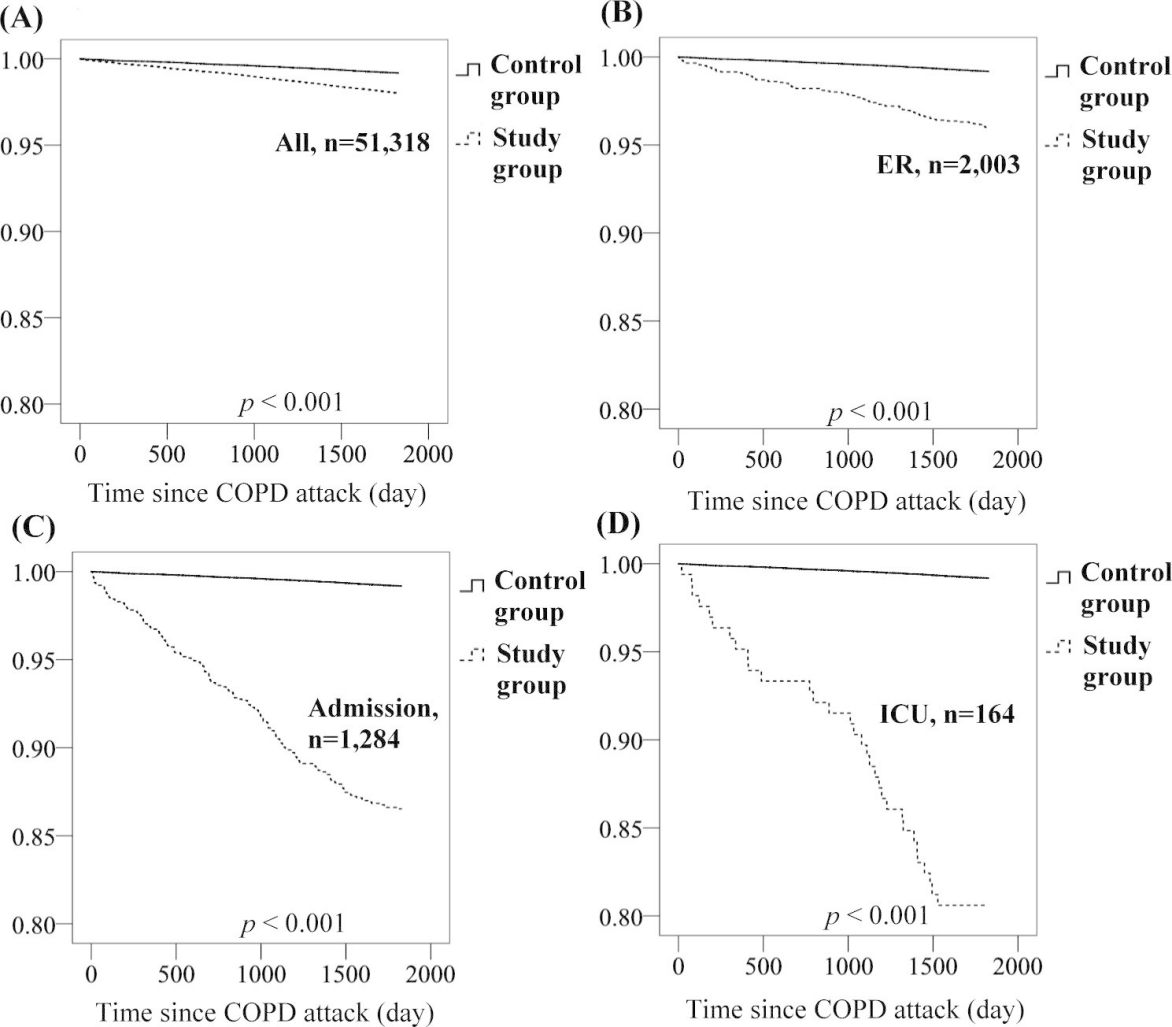
Dementia-free survival curves for (A) the entire study group and those with different severities of AEs of COPD, (B) those who visited only the ED, (C) those who were admitted to the hospital, (D) and those who were admitted to the ICU. More severe AEs of COPD indicated a higher risk of developing dementia (all p < 0.001)

The dementia-free survival curves for the study and control groups are shown in Fig. 1. More severe AEs of COPD indicated a higher risk of developing dementia (all p < 0.001).

## Discussion

Although the link between COPD and dementia has been reported in some previous studies, the severity of acute exacerbations and treatments for COPD that would increase (or decrease) the risk of developing dementia have yet to be well addressed. Laura Ranzini et al. reported that the severity of COPD, poorer quality of life, smoking, and lower socioeconomic status were all associated with cognitive impairment or dementia.[35] In this study, we demonstrated that medications and the different severities of AEs of COPD were important risk factors for the occurrence of dementia. In this study, we noted that the COPD group had a crude HR 2.51 times greater than that of the control group. Furthermore, even after adjusting for the demographics and baseline comorbidities of patients, the HR of dementia in the COPD group was still higher. In the control group, the “overall rate” of dementia diagnosis was only 0.8%. However, among people aged over 70 years in the control group, the dementia rate was 8.6% (n = 360). The data are similar to those of another dementia study among Taiwanese individuals.[36] That study concluded that the overall incidence of dementia (for people aged over 65 years) was 8.0%. Therefore, our sample was representative.

The Initial severity classification in the ED was a strong risk factor for developing dementia. Some previous studies have reported that the characteristics of dementia (including vasculopathy, inflammatory response and hypoxemia damage) were more obvious in the central nervous system of severe COPD patients.[37, 38] Prolonged or chronic hypoxia could alter the dysregulation of calcium ion homeostasis and induce neuroinflammation (leading to chronic activation and recruitment of proinflammatory immune cells).[27, 28] Therefore, we suspect that more severe AEs might be related to worse hypoxia and further induce neuronal dysfunction. In addition, Xuecai Fu et al. mentioned that delirium was more common among severe COPD patients, especially among those admitted to the ICU or who required mechanical ventilation (related to abnormal function of neurotransmitters, damage of the blood–brain barrier and ICU syndrome).[39] Some of these studies further noted that severe COPD was a risk factor for developing dementia. Most elderly patients choose to visit the ED for critical conditions or initial airway management.[40, 41] However, the association between severity classification upon ED visit for an acute attack and the occurrence of new-onset dementia has not been well addressed. In this study, we focused on patients with AEs of COPD who visited the ED, and we found that the severity of acute presentations was a strong risk factor for subsequent dementia occurrence. We further found that ED patients who required hospital or ICU admission were clearly at higher risk than ED-treated patients and the whole COPD cohort. Furthermore, ICU admission might be associated with a higher risk for dementia.

We demonstrated that bronchodilators (beta-2 agonists, muscarinic agonists and xanthine derivatives) may be associated with a lower risk of dementia. There were three possible major reasons for this finding. First, bronchodilators can increase oxygenation function by improving the essential symptoms or respiratory status of COPD.[42, 43] Second, bronchodilators have a stimulating effect on the central nervous system. Furthermore, long-acting beta-2 agonists have been shown to inhibit microglial activation, thus decreasing neurotoxicity in Parkinson’s disease.[15, 40] Finally, bronchodilators can decrease the level of glutamate (acting as endogenous neurotoxins in the glutamatergic system in the pathophysiology of dementia) via astrocyte activation.[3, 15] Some reasons might influence the percentage of patients with long-term bronchodilator use. First, our cohort might have had mild disease, and thus they did not require long-term treatment. Second, some of the COPD patients might have been asthma patients (miscoding). Long-term use of bronchodilators among COPD patients might be associated with dementia; however, future studies should investigate the potential therapeutic relationship more rigorously. Finally, antibiotics are effective in treating acute exacerbations of COPD and can reduce the frequency of exacerbations[20, 21]. In comparisons of the unadjusted HR of dementia (HR: 2.51, 95% CI: 2.24–2.81), whether patients received long-term or short-term medication treatment was associated with a lower risk of dementia. Compared to COPD, asthma might be more common in younger populations. These patients are more likely to have a lower risk of dementia. COPD and asthma are considered different disease entities; however, they could have very similar presentations.[44] Several studies have even noted that “asthma-chronic obstructive pulmonary disease overlap syndrome” (ACOS) might be present in 25 to 37% of new COPD patients.[45, 46] The effect of bronchodilators in the association with a lower risk of dementia might be related to miscoded asthma cases.

## Limitations

This study had some limitations. Our study obtained data from a government deidentified secondary database using ICD-9-CM codes, which are less specific in identifying health conditions compared to ICD-10-CM codes (a natural limitation). Regarding our exclusion criteria, some patients with hydrocephalus might not have had impaired brain cognitive function. Excluding patients with hydrocephalus might have caused the sample size to be limited. There are diseases such as asthma that as are treated mainly with bronchodilators, such as COPD. However, no similar data in relation to dementia were researched in this study. It is important to differentiate the bronchodilator effect from COPD disease itself considering the pathophysiology of dementia. Moreover, many types of bronchodilators are included in the treatment of COPD, but our research lacked detailed data to analyze the relationship of each with dementia. Another limitation was the risk of underdiagnosis of dementia (we relied on a database and did not perform our own diagnostic tests). In addition, the effect of bronchodilators, which are associated with lower risks of dementia, might have been related to miscoded asthma cases. The database lacked clinical laboratory data, smoking status information and detailed results of examinations of COPD (i.e., arterial blood gas, carbon dioxide retention, pulmonary function tests, data on forced vital capacity and hypoxia), which can be key risk factors for dementia. Finally, we assumed that prescriptions of medications equated to patients taking the medications and that we were unable to assess adherence.

In conclusion, bronchodilator administration might be associated with a decreased risk of developing dementia. More importantly, patients who suffered AEs of COPD and initially visited the ED and required ICU admission had a higher risk of developing dementia.

## Electronic supplementary material

Below is the link to the electronic supplementary material.

Supplementary Material 1

## Data Availability

All datasets generated for this study are included in the article.
