# Supplementary material for: The adjusted impact of different severities of acute exacerbations and medications on the risk of developing dementia in COPD patients
Source: BMC Pulm Med. 2023 Mar 29;23:103. doi: 10.1186/s12890-023-02386-8 (PMC10061783; doi:10.1186/s12890-023-02386-8)
Supplement: Supplementary file 1 — Supplementary Material 1 [file 12890_2023_2386_MOESM1_ESM.docx]

**Additional file 1**


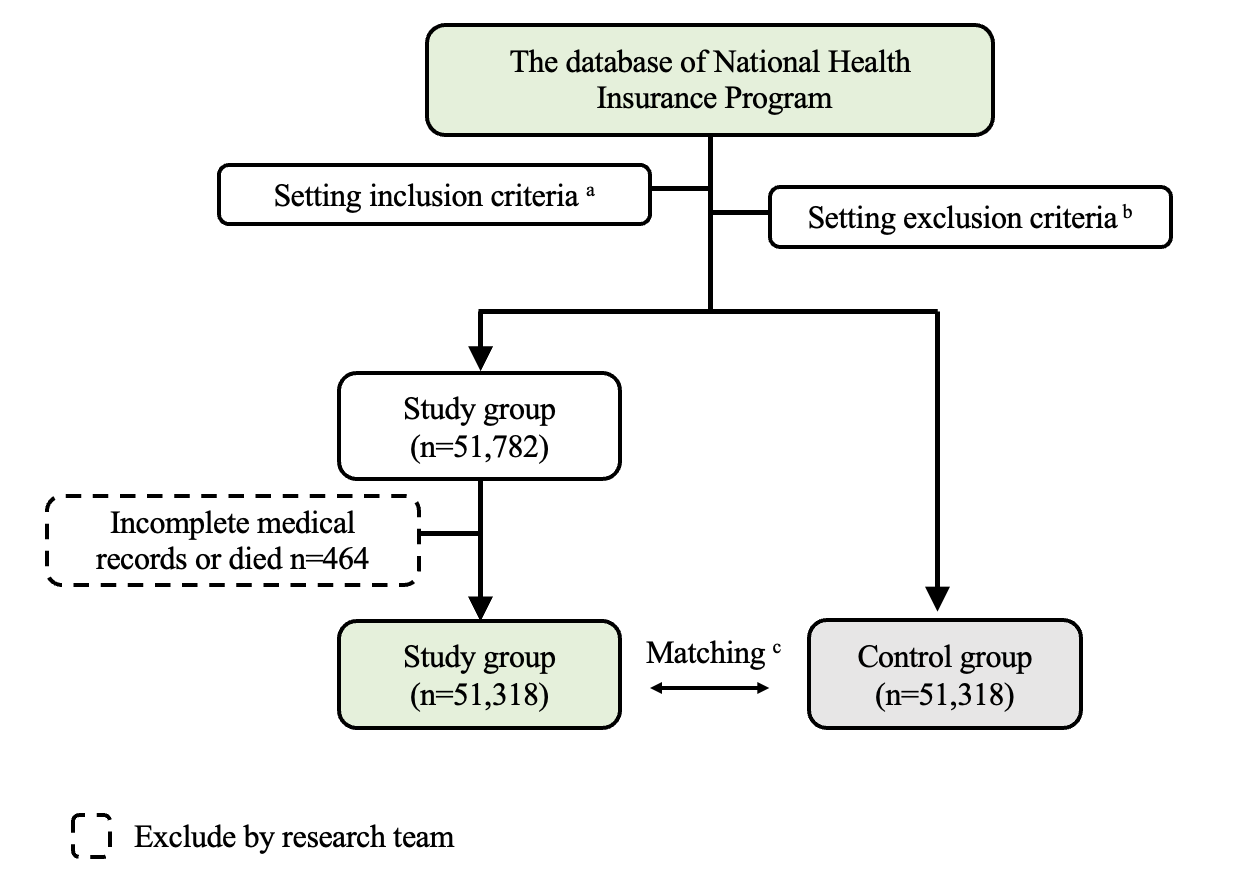


**The flowchart of exclusion**. Before the study starts, we need to provide proposal and input the inclusion and exclusion criteria to the government’s database. The database would directly output the final data which we asked. Therefore, how many patients were excluded during the “program processing” were not well known. Once we received the data, we checked it first, if data were incomplete (or patient died), we excluded them from study (n=464). The detail information of inclusion^a^, exclusion^b^, and matching^c^ are shown in the section of method of main text.
